# Mutation of *BAM2* rescues the *sunn* hypernodulation phenotype in *Medicago truncatula*, suggesting that a signaling pathway like CLV1/BAM in *Arabidopsis* affects nodule number

**DOI:** 10.3389/fpls.2023.1334190

**Published:** 2024-01-11

**Authors:** Jacklyn Thomas, Julia Frugoli

**Affiliations:** Department of Genetics and Biochemistry, Clemson University, Clemson, SC, United States

**Keywords:** nodulation, meristems, autoregulation of nodulation, *M. truncatula*, BAM, SUNN

## Abstract

The unique evolutionary adaptation of legumes for nitrogen-fixing symbiosis leading to nodulation is tightly regulated by the host plant. The autoregulation of nodulation (AON) pathway negatively regulates the number of nodules formed in response to the carbon/nitrogen metabolic status of the shoot and root by long-distance signaling to and from the shoot and root. Central to AON signaling in the shoots of *Medicago truncatula* is SUNN, a leucine-rich repeat receptor-like kinase with high sequence similarity with CLAVATA1 (CLV1), part of a class of receptors in *Arabidopsis* involved in regulating stem cell populations in the root and shoot. This class of receptors in *Arabidopsis* includes the BARELY ANY MERISTEM family, which, like CLV1, binds to CLE peptides and interacts with CLV1 to regulate meristem development. *M. truncatula* contains five members of the *BAM* family, but only *MtBAM1* and *MtBAM2* are highly expressed in the nodules 48 hours after inoculation. Plants carry mutations in individual *MtBAM*s, and several double *BAM* mutant combinations all displayed wild-type nodule number phenotypes. However, *Mtbam2* suppressed the *sunn-5* hypernodulation phenotype and partially rescued the short root length phenotype of *sunn-*5 when present in a *sunn-5* background. Grafting determined that *bam2* suppresses supernodulation from the roots, regardless of the *SUNN* status of the root. Overexpression of *MtBAM2* in wild-type plants increases nodule numbers, while overexpression of *MtBAM2* in some *sunn* mutants rescues the hypernodulation phenotype, but not the hypernodulation phenotypes of AON mutant *rdn1-2* or *crn*. Relative expression measurements of the nodule transcription factor MtWOX5 downstream of the putative *bam2 sunn-5* complex revealed disruption of meristem signaling; while both *bam2* and *bam2 sunn-5* influence *MtWOX5* expression, the expression changes are in different directions. We propose a genetic model wherein the specific root interactions of BAM2/SUNN are critical for signaling in nodule meristem cell homeostasis in *M. truncatula*.

## 1 Introduction

Legumes tightly control nitrogen-fixing symbioses leading to nodulation. The autoregulation of nodulation (AON) pathway negatively regulates the number of nodules formed in response to the metabolic status of the shoot (carbon) and root (nitrogen) (reviewed in Ferguson et al., 2019; Chaulagain & Frugoli, 2021). Genetic analysis has identified multiple genes that when mutated cause plants to hypernodulate, evidence of a network of regulation in AON from both the root and the shoot (reviewed in Roy et al., 2020).

In AON, very early after rhizobial infection, expression of a subset of genes encoding the CLAVATA3/Embryo Surrounding Region (CLE) peptides, *MtCLE12* and *MtCLE13* in *Medicago truncatula*, is induced (Mortier et al., 2010). In *Lotus japonicus*, a similar increase upon infection is observed in the LjCLE-root signaling (RS)1, LjCLE-RS2, and LjCLE-RS3 peptide-encoding genes (Okamoto et al., 2009; Nishida et al., 2016; Hastwell et al., 2017). These CLE peptides are 12–13 amino acids in length and are signaling peptides derived from the C-terminal region of pre-proproteins (Araya et al., 2016; Yamaguchi et al., 2016). In *Arabidopsis thaliana*, CLE function is associated with regulating cell proliferation and differentiation during development, especially in the shoot and root apical meristems. In *M. truncatula*, the MtCLE12 peptide has been shown genetically to be modified by an enzyme from the hydroxyproline *O*-arabinosyltransferase (HPAT) family encoded by the *M. truncatula ROOT DETERMINED NODULATION1* (*RDN1*) gene (Kassaw et al., 2017); the mutation of *RDN1* produces a supernodulation phenotype (Schnabel et al., 2011). After nodulation-suppressing CLE peptides are processed, they travel through the xylem to the shoot where they are perceived by a homo- or heterodimeric receptor complex, likely in the parenchyma cells of the vasculature (Kassaw et al., 2017).

Central to this receptor complex is a shoot-acting leucine-rich repeat (LRR) RLK, known as SUPER NUMERARY NODULES (SUNN) in M. truncatula, HYPERNODULATION ABBERANT ROOT FORMATION (HAR1) in L. japonicus, NODULE AUTOREGULATION RECEPTOR KINASE (NARK) in Glycine max, and SYMBIOSIS29 (SYM29) in Pisum sativum (Krusell et al., 2002; Searle et al., 2003; Schnabel et al., 2005). Mutations in the MtSUNN gene produce a hypernodulation phenotype and altered root development (Schnabel et al., 2005). A shoot-controlled increase in nodulation is also observed in the MtCORYNE Tnt1 insertion mutant (crn) of M. truncatula (Crook et al., 2016), as well as the klv mutant (Oka-Kira et al., 2005) and the clv2 mutant (Krusell et al., 2011) in L. japonicus. Bimolecular fluorescence analysis showed that SUNN forms heteromomers with homologous CLV1-interacting proteins CLAVATA2 (CLV2), CORYNE (CRN), and KLAVIER (KLV) (Oka-Kira et al., 2005; Miyazawa et al., 2010; Krusell et al., 2011; Crook et al., 2016). Genetic studies of a hypernodulating mutant in L. japonicus identified a root-responsive gene TOO MUCH LOVE (TML) that regulates nodule numbers in the root from shoot-derived signaling (Magori et al., 2009; Takahara et al., 2013). Knockdown of two TML genes, MtTML1 and MtTML2, showed increased nodule number, suggesting a role for these genes in AON (Gautrat et al., 2019).

Leucine-rich repeat receptor-like kinases (LRR-RLKs) are important in meristem development in most land plants. The similarity between symbiotic AON receptors and their *Arabidopsis* meristem regulating counterparts like RPK2 (Kinoshita et al., 2010) suggests that like the shoot apical meristem (SAM) and root apical meristem (RAM), LRR-RLK complexes are involved in AON signaling to nodule meristems (Krusell et al., 2002; Crook et al., 2016). Some (but not all) legume *CLV2* and *RPK2-*related mutants show defects in the SAM, but the *sunn Atclv1*-related mutants do not have SAM defects, indicating overlapping receptor complexes controlling SAM activity and nodule numbers in legumes (Krusell et al., 2002; Schnabel et al., 2005).

Since similar molecules regulate shoot, root, and nodule meristems, we wondered if BAMs, which have not yet been linked to AON in any legume, could be involved in the regulation of the nodule meristem. The *Arabidopsis* BAMs bind to a wider range of CLE peptides and show more diverse expression patterns compared to CLV1 (reviewed in Yamaguchi et al., 2016). We reasoned that studying *BAM* expression in *M. truncatula* could expand our understanding of plant receptor interacting partners’ expression levels and location effects during signal transduction pathways. Since AtCLV1 forms a complex with AtBAMs, MtSUNN might form a complex with MtBAMs to control nodule numbers. Mutational and overexpression experiments of the *M. truncatula BAM* gene family reported below support a model in which the genetic interaction of BAM2 and SUNN provides a signal to limit nodulation.

## 2 Materials and methods

### 2.1 Phylogenetic analysis

The tree was constructed with MEGA X using the maximum likelihood method (Kumar et al., 2018) with bootstrap replicates n = 1,000. The analysis involved 21 amino acid sequences summarized in **Supplementary Table 1** for a total of 1,107 positions in the final dataset, with AtCLV1 used to root the tree.

### 2.2 Mutant line screening

The *Tnt-1* mutant lines were isolated from populations of insertion lines obtained from the Nobel Foundation Medicago Mutant Database now located at https://medicagomutant.dasnr.okstate.edu/mutant/index.php described in Tadege et al. (2008). The following *Tnt-1* mutant pools were used in this study: *Mtbam1* (NF2153), *Mtbam2* (NF7126), *Mtbam3* (NF2071), *Mtbam4* (NF2835), *Mtbam5* (NF2488), and *sunn-5* (NF2262). Initial PCR screening from pools of *Tnt1* insertions in *M. truncatula BAM*s was carried out to identify plants carrying the insertion in a BAM gene using PCR from genomic DNA. Two primer sets for each gene were designed specifically for capturing the wild-type allele and another specific to flanking regions of the *Tnt1* insert and *Tnt1* forward primer (see **Supplementary Table 2**). Plants identified as carrying an insertion were selfed, and the next generation was screened using PCR to obtain single homozygous mutants for all five *BAM*s from the *Tnt-1* pools. Each homozygous mutant was backcrossed to the parental R108 ecotype and reisolated, following the inheritance of the insertion via PCR.

### 2.3 PCR to identify single and double mutants and *sunn-5* structure

A leaf press was made by pressing a leaflet of each plant to a Plant Card (Whatman™, GE Healthcare UK Limited, Amersham, UK) according to the manufacturer’s instructions for long-term storage. A 1.2-mm-diameter piece of Plant Card was excised and washed with Whatman™ FTA Purification Reagent (GE Healthcare UK Limited, UK) followed by TE-1 buffer according to the manufacturer’s instructions and directly used in PCR. The 10-μL PCR used to identify single and double mutants contained 2 μL of gDNA (equivalent to 100 ng RNA), 1 mm each primer, 0.2 mm dNTPs, 1× colorless Go*Taq* buffer, and 1 U Go*Taq* (Promega, Madison, WI, USA) with cycling conditions of 95°C for 2 min followed by 40 cycles of 95°C for 5 sec, 55°C for 10 sec, and 72°C for 20 sec. Sequencing of *sunn-5* was performed using PCR products amplified from cDNA using pairs of gene-specific primers for sequences JF7092, JF7093, and JF7094 and a *Tnt-1* specific primer for JF7091 as referred to in **Supplementary Figure 1**. PCR conditions were 95°C for 2 min followed by 35 cycles of 95°C for 30 sec, 55°C for 30 sec, and 72°C for 4 min. Sequencing was performed by Arizona Life Sciences.

### 2.4 Plant crossing

Two pairs of fine-tip forceps (HL-14 #5, Buy in Coins Promotion) and straight-edge scalpels (scalpel blade handle 9303 #3 and scalpel blade 9311 #11, both from Microscopes America, Cumming, GA, USA) were used for keel petal incision and the removal of anthers from the unopened female flower bud and artificial cross-pollination (Veerappan et al., 2014). The mature pods from the successful cross-pollinations were wrapped using micro-perforated polythene sheets (MP1120160T, Prism Pak, Berwick PA, USA) to dry on the plant before harvesting at desiccation. The following crosses were successfully performed and the F2 was screened for double mutants: *bam2* × *bam3*, *bam2* × *bam4*, *bam5* × *bam4*, *bam2* × *sunn-5*, *bam3* × *sunn-5*, and *bam3* × *sunn-5*.

### 2.5 Plant growth conditions and materials

The identified homozygous *bam* mutants used for making genetic crosses were grown in a greenhouse, with supplemental lighting to create a 14:10 light:dark (L:D) cycle, a nightly minimum of 18°C, and a daily maximum between 21°C and 27°C. For nodulation screening, the following seeds were used: *M. truncatula* wild-type Jemalong A17; the AON-defective mutants *rdn1-2* (Schnabel et al., 2011), *sunn-1*, and *sunn-4* (Schnabel et al., 2005); and *M. truncatula* wild-type R108 and *Tnt1* mutants *bam1-5* (this work), *crn* (Crook et al., 2016), and *cra2* (Huault et al., 2014) in the R108 genetic background (Garmier et al., 2017). Seeds were removed from the pods and scarified in sulfuric acid for 8 min, rinsed five times in dH_2_O, imbibed in dH_2_O for 2–3 hours at room temperature, then placed at 4°C for 48–72 hours in the dark to synchronize germination, and allowed to germinate for 1 day at room temperature. This procedure as well as the growth in an aeroponic apparatus is described in Cai et al. (2023). Inoculation of all whole plants screened for nodulation phenotypes was carried out using *Sinorhizobium meliloti* RM41 (Luyten and Vanderleyden, 2000) for the R108 ecotype with 6 mL each of an OD_600_= 0.2 culture, and data were collected 14 days post inoculation (dpi). The apparatus was subjected to 14:10 L:D conditions and maintained at room temperature. Nodule number comparisons of wild-type R108 plants and hypernodulating *sunn-5* plants to individual single *bams* plants, *bam* double-mutant plants, and *bam sunn-5* plants were carried out by visual count of individual plant roots using head-mounted magnification glasses.

Transgenic hairy root plants made with *Agrobacterium rhizogenes* containing the gene constructs described below or the empty vector were grown in TY (selection 25 μg/mL kanamycin) and used for hairy root transformation as described in Limpens et al. (2004). Inoculated seedlings were grown on plates containing modified HMF (Huo et al., 2006). The plates were incubated flat in the growth chamber at 20°C for 3 days to facilitate transformation and then moved to 25°C to facilitate root growth for 2–3 weeks. Transformed roots expressing the DsRED fluorescence marker were identified on the Olympus SZH-10 stereoscopic system (excitation 540 nm; emission detected at 570–625 nm), and untransformed roots were removed. The plants with transformed roots were then grown in perlite for 2 weeks before being used for the nodulation assay described in Kassaw et al. (2017) by inoculation using *Sinorhizobium medicae* strain ABS7 (Leong et al., 1985; Bekki et al., 1987) for A17 plants or *S. meliloti* RM41 as described in Kassaw and Frugoli (2012). Nodules were counted at 14 dpi.

Grafting was performed following the protocol of Kassaw and Frugoli (2012), and grafted plants were allowed to grow in the growth chamber at 20°C with a 16-hour photoperiod for 3 days to facilitate transformation and then moved to 25°C with a 16-hour photoperiod to facilitate root growth for 2–3 weeks. Plants were transferred to pots filled with washed, autoclaved perlite and maintained on a light bench with (14:10 L:D) cycle and temperatures between 21°C and 24°C. Plants were watered daily with a 100-fold dilution of water-soluble 20:10:20 (N:P: K) Peat-Lite Special fertilizer (Scotts Company, Marysville, OH, USA) for 5 days. After an additional 4 days of nutrient starvation induced by watering with water alone, the plants were used for inoculation experiments using *S. medicae Rm41*, and nodule number was assessed at 14 dpi. The survival rate for all grafted plants was approximately 40%–50% except for *sunn-5* shoot-to*-sunn-5* root plants, which was approximately 10%–20%. The reduced survival of *sunn* self-grafts is common in both our laboratory and others due to disrupted auxin homeostasis in *sunn* mutants (van Noorden et al., 2006).

#### 2.5.1 Creation of *BAM* overexpression constructs

The *MtBAM1*, *MtBAM2*, and *MtBAM3* sequences were amplified from *M. truncatula* R108 cDNA using overlap PCR with the primers listed in **Supplementary Table 2**. Using overlap extension PCR allowed for the cloning of large fragments by fusing two gene halves together to generate large transcripts for cloning (Simionatto et al., 2009). Each transcript was amplified using the following PCRs (10 μL) that contained 2 μL of cDNA (equivalent to 100 ng RNA), 1 mm of each primer, 0.2 mm dNTPs, 1× colorless Go*Taq* buffer, and 1 U Go*Taq* (Promega, Madison, WI, USA) with cycling conditions of 95°C for 2 min followed by 40 cycles of 95°C for 5 sec, 55°C for 10 sec, and 72°C for 20 sec. PCR products were gel extracted and purified using the Zymoclean gel DNA recovery kit (Zymo Research, Irvine, CA, USA). The PCR products were then digested with *Kpn*I and *Spe*I restriction enzymes and ligated into the *Kpn*I and *Spe*I sites of the pCDsRed/35S vector (Karimi et al., 2002) under the control of the 35S promoter using *Escherichia coli DH5α* cells. The construct was confirmed by sequencing and transferred to *A. rhizogenes* strain *ArQUA1* (Quandt and Hynes, 1993) by electroporation for use in hairy root transformation.

### 2.6 *A. rhizogenes-*mediated plant transformation

Seedlings were transformed as described by Limpens et al. (2004). The hypocotyls of 5-day-old seedlings were cut and transformed by lightly scraping on the surface of Luria–Bertani plates densely grown with *A. rhizogenes* strain *ArQUA1* (Quandt and Hynes, 1993) containing the appropriate binary vector and antibiotic selections at 30°C for 48 hours. After 5 days of cocultivation with the *A. rhizogenes* in the growth chamber at 23°C at 16:8 L:D, the seedlings were transferred to a nutrient-rich hairy root emergence medium (Limpens et al., 2004) containing 300 µg/mL cefotaxime (Phytotechnology Laboratories, St. Lenexa, KS, USA) sandwiched between two half-round Whatman filter papers grown under the same growth conditions. Five days later, the top filter papers were removed from the plates, and the seedlings were allowed to grow for an additional 5 days on the same emergence medium placed vertically in the same growth chamber. Transformed roots expressing the DsRED fluorescence marker were identified on Olympus SZH-10 stereoscopic system (excitation 540 nm; emission detected at 570–625 nm), untransformed roots were removed as described in Kassaw and Frugoli (2012), and plants were transferred to perlite. For acclimation to perlite, plants were watered for 5 days with a 100-fold dilution of water-soluble 20:10:20 Peat-Lite Special fertilizer (Scotts). Fertilization was then withdrawn, and the plants were hydrated with water alone for an additional 5 days to induce the nitrogen deficiency required for nodulation. Plants were then inoculated with *S. meliloti* RM41 or *S. medicae* ABS7 according to ecotype, with 6 mL each of an OD_600_= 0.2 culture, and data were collected at 14 dpi.

### 2.7 Quantitative real-time PCR

For RNA extraction, the nodule-forming zone described in Schnabel et al. (2023a) was harvested from 10 plants for each genotype (*A17*, *sunn-1*, and *sunn-4*, *rdn2-1*, *cra2*, *R108*, *bam2*, *bam2 sunn-5*, and *sunn-5*) at multiple time points (0 hours, i.e., prior to inoculation, 12 hours post inoculation (hpi), and 48 hpi) and stored at −80°C. RNA was isolated from nodulating roots using the E.Z.N.A. Plant RNA Kit (Omega Bio-Tek, GA, USA) according to the manufacturer’s instructions. Each RNA sample was digested with RNase-free DNase (Promega) treatment for 40 min to remove genomic DNA contamination. The iScript cDNA Synthesis Kit (Bio-Rad, Hercules, CA, USA) was used to synthesize cDNA from 1 μg of RNA in a 20-μL reaction. The cDNAs were then diluted to 40 μL. All experiments were performed using iTaq™ Universal SYBR® Green Supermix (Bio-Rad, CA, USA) and the Applied Biosystems QuantStudio 3 Real-Time PCR System. Each reaction was performed in triplicate, and results were averaged for a single biological replicate. The total reaction volume was 12.5 μL (5 μL of master mix including 0.5 μL of each primer [0.5 μM final concentration] and 2.0 μL of cDNA). Cycle threshold values were obtained using the QuantStudio 3 software, and expression was determined relative to the *internal reference PI4K* (see below). Intron spanning primers unique in the *M. truncatula* genome based on National Center for Biotechnology Information Primer-BLAST analysis were used (**Supplementary Table 2**). Relative expression levels of genes were assayed using the Pfaffl method (Pfaffl, 2001) relative to previously validated housekeeping reference gene phosphatidylinositol 3- and 4-kinase belonging to ubiquitin family (PI4K; Medtr3g091400 in MtV4.0) (Kakar et al., 2008). Data from the three biological replicates were used to estimate the mean and standard error.

### 2.8 Statistical analysis

Statistical difference analysis for pairwise mean comparison was calculated by the Tukey–Kramer honestly significant difference (HSD) using JMP Pro 13.1 (https://www.jmp.com/en_us/home.html). For qPCR, absolute quantification was used to compare the quantification cycle (Cq) values of test samples to those of standards (*PIK* internal reference) of known quantity plotted on a standard curve. The quantity was normalized to a unit amount of nucleic acid (i.e., concentrations). The statistical analysis tests for the probability that the relative expression RE ≠ 1 for data normalized to control.

## 3 Results

### 3.1 Phylogenetic and structural analysis of BAM proteins

**Figure 1:**
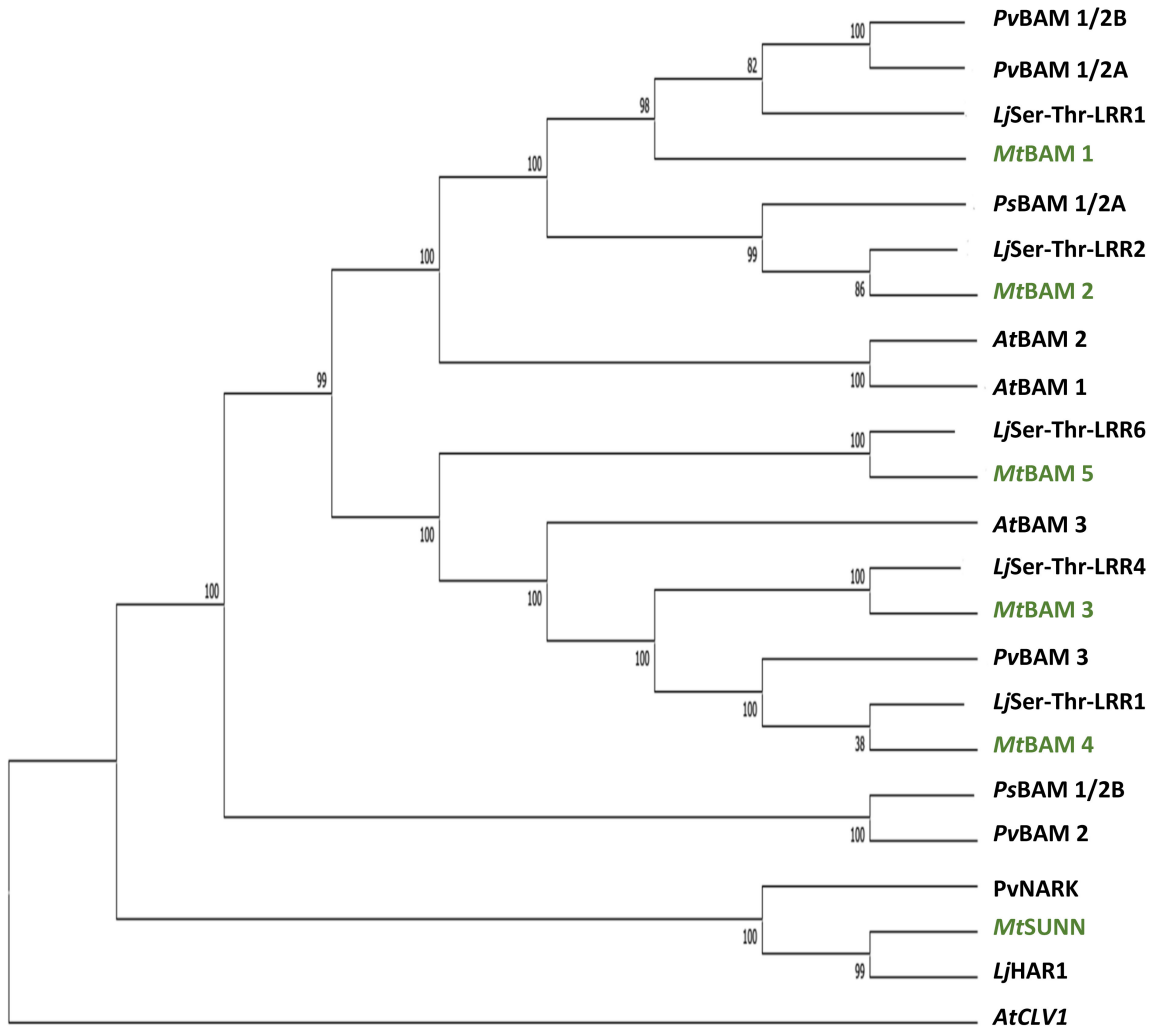
Relatedness of BAM receptor kinases between legumes and *Arabidopsis*. Phylogenetic tree created using the maximum likelihood method and rooted with *At*CLV1, with branches supported by at least 80% of the bootstrap replicates (*n* = 1,000). *Medicago truncatula* BAM proteins are highlighted in green. Accession numbers for genes in tree are in **Supplementary Table 1**.

**Figure 2:**
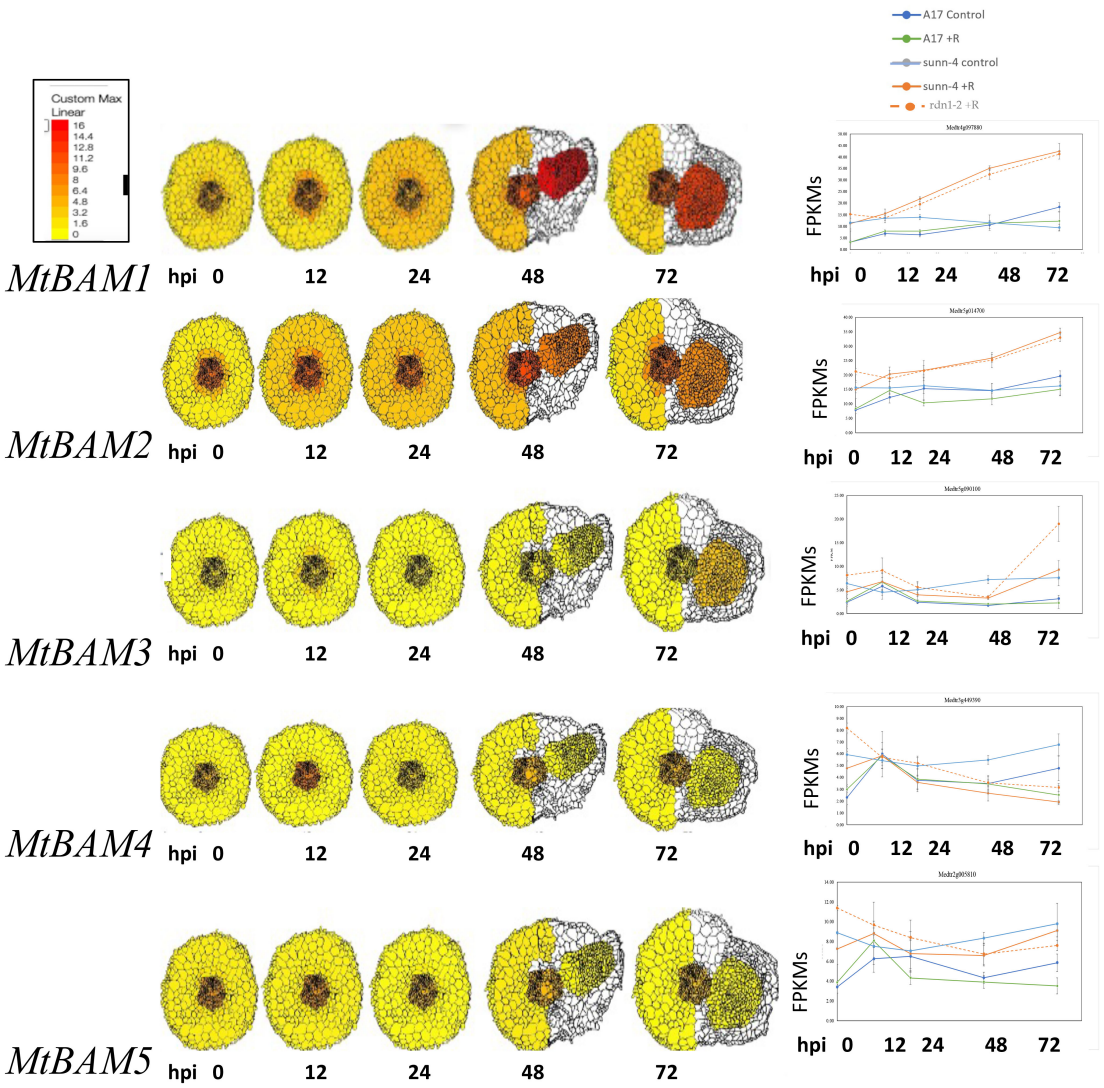
*MtBAM* expression in individual tissues and whole root segments during nodule formation. The tissue-specific expression patterns on the left for each *MtBAM* were created using ePlant and the data in Schnabel et al. (2023b) and a fixed maximum for comparison between genes across a time course at 0 to 72 hpi. The whole root expression traces for each *MtBAM* on the right are from the same time course and conditions, taken from the data in Schnabel et al. (2023a). Expression (FPKMs) of BAMs from 0 to 72 hpi are displayed as blue = A17 control, green = A17 + rhizobia, light blue = *sunn-4* control, orange = *sunn-4* + rhizobia, and orange dotted = *rdn1-2* + rhizobia.

A phylogenetic analysis by neighbor-joining was performed between the protein sequences of legume and *Arabidopsis* BAMs, with CLV1 and the related symbiotic kinases used to root the tree (**Figure 1**). There was not a one-to-one relationship between MtBAMs and AtBAMs; rather, they shared similarities with BAMs from other legume BAM-Like LRR kinases and *Arabidopsis*. For all the legumes, however, there were two BAM proteins with similarities to *At*BAM1 and *At*BAM2, including *Mt*BAM1 and *Mt*BAM2. The *Mt*BAM1 and *Mt*BAM2 proteins have 80% sequence similarity between them, while *Mt*BAM1 has 81% similarity to *At*BAM1, the highest shared similarity among all comparisons*. Mt*BAM3, *Mt*BAM4, and *Mt*BAM5 were closer phylogenetically to the *At*BAM3 protein with a 55%–56% similarity for each. The branching structure suggests that the five BAM genes in legumes arise from duplications of a BAM3 ortholog (**Figure 1**). To further pursue the idea that these duplicate genes might be specific for nodulation, the expression levels of each *MtBAM* in individual root tissues were examined during nodule development. Tissue level data from a Laser Capture Microdissection experiment (Schnabel et al., 2023b) and root segment transcriptome data from an unfixed roots experiment (Schnabel et al., 2023a) were examined, following nodule development at 0, 12, 24, 48, and 72 hpi (**Figure 2**). *MtBAM1* and *MtBAM2* were expressed in nodule meristems at 48 and 72 hpi at higher levels than the other *BAM* family members. *MtBAM1* and *MtBAM2* expression at 12 and 24 hpi was higher in the inner cortical cells at the xylem pole, the cells from which nodules arise (Libbenga and Harkes, 1973). In addition, *MtBAM1* and *MtBAM2* had higher expression in nodule meristems at 48 and 72 hpi compared to *MtBAM3*, *MtBAM4*, and *MtBAM5*. *MtBAM3*, *MtBAM4*, and *MtBAM5*; all displayed similarly low levels of expression throughout nodule formation, with no clear signature in an individual tissue in this experiment (**Figure 2**). The expression data suggest that *MtBAM1* and *MtBAM2* are most likely to be involved in early nodule formation.

### 3.2 Root length and nodule number phenotypes of *MtBAM* mutants are wild type

With the expression data in hand, we generated single homozygous mutants for the *BAM* genes in *M. truncatula* using PCR to follow the segregation of *Tnt1* insertions in lines isolated from pools of plants from the Nobel Foundation Medicago Mutant Database identified as containing an insertion in an *MtBAM* gene. The progeny of plants determined to be homozygous for a *Tnt-1* insertion in each *BAM* were tested for nodule number on an aeroponic chamber (see Materials and Methods). All the single *MtBAM* mutants had *Tnt1* insertions in approximately the same place in the LRR domains, and wild-type nodule numbers (**Supplementary Figure 2A**). Since other nodule regulatory mutants also have root length defects (Schnabel et al., 2005; Schnabel et al., 2010; Schnabel et al., 2011), single mutant plants were grown in the absence of rhizobia, and root length was measured at the equivalent development point to 14 dpi. None of the single *MtBAM* mutants displayed a root length phenotype different from the wild-type plants (**Supplementary Figure 2B**). Similar to the observation of no meristem defects in single *bam* mutants in *Arabidopsis* (DeYoung et al., 2006), no nodule number or root length phenotype was observed in the *M. truncatula* single *BAM* mutants. We also generated double mutants of *Mtbam2* with *Mtbam3* and *Mtbam4*, as well as an *Mtbam4;Mtbam5* double mutant, to determine if an effect is observable with the loss of more than one *BAM*, as is the situation with *Arabidopsis* mutants (DeYoung and Clark, 2008), but these also had no effect on nodule number or root length (**Supplementary Figure 3**). Interestingly, we were unable to obtain seeds from any cross of the *Mtbam1* mutant to any other *Mtbam* mutant, despite multiple attempts. Examining the ePlant database (Waese et al., 2017), transcriptomic data for *MtBAM1* show high levels of expression in tissues such as pods, flowers, stems, and developing seeds (Benedito et al., 2008). When attempting to make crosses with *bam1*, the stamen looked frail and opaque with few pollen granules visible (data not shown), and this may explain the lack of success generating *bam1 bamx* double mutants.

### 3.3 *Mtbam2* suppresses the *sunn-5* supernodulation phenotype in the double mutant

**Figure 3:**
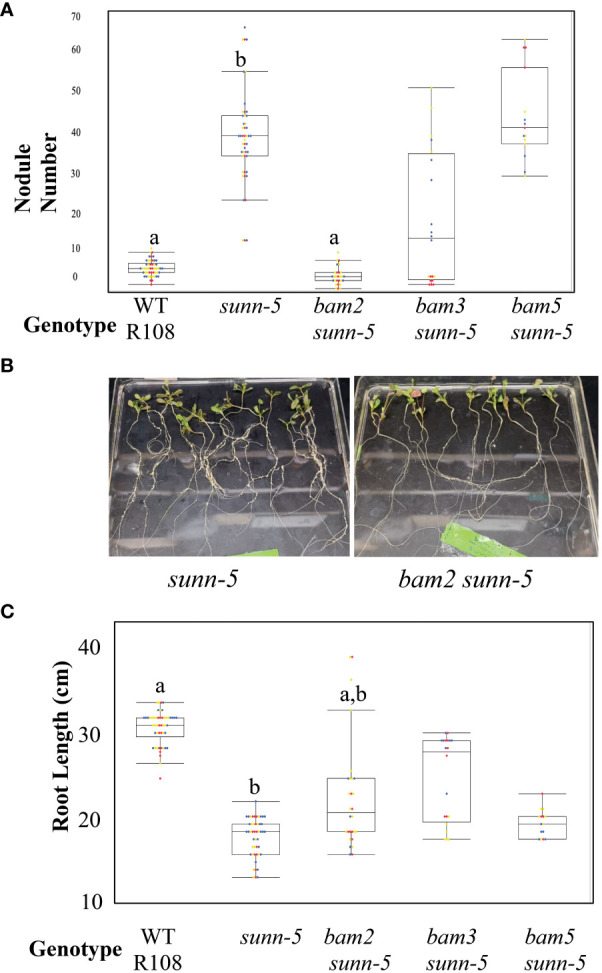
*bam2* suppresses nodule number in *sunn-5* mutants, while *bam2* and *bam3* suppress root length defects. **(A)** Nodule number per plant at 14 dpi. Letters denote significance compared to wild-type **(B)** photos of *sunn-5* and *bam2 sunn-5* plants. **(C)** Root length in wild-type plants compared to *sunn-5* and *bam* double mutants at 14 days without rhizobia. Letters denote significance compared to wild type. Dots represent individual plants. Each dot color is associated with independent biological replicates: rep1 = red, rep2 = yellow, and rep3 = blue. Box plot mean is indicated with a line, while whiskers are ± standard error, pairwise comparison Tukey–Kramer honestly significant difference (HSD), p-value a:b > 0.001. p-value a:a,b > 0.001.

Since *Arabidopsis BAM*s were originally isolated by the effects observed in a *clv1* mutant background (DeYoung et al., 2006), we reasoned that mutations in *MtBAM* genes might only have noticeable effects on nodule number in a *sunn* mutant background. We created a set of *bam*/*sunn* double mutants by crossing and isolating F2 progeny using PCR confirmation of the phenotype: *bam2 sunn-5*, *bam3 sunn-5*, and *bam5 sunn-5*. We observed a suppressive effect on nodule number in the *bam2 sunn-5* double mutant (**Figure 3A**). We applied a slightly different observation to the root length phenotype; the *sunn-5* short root phenotype was partially rescued by *bam2* or *bam3*, but not *bam5* (**Figure 3B**). Because of the suppressive effect of *bam2* on both phenotypes of *sunn-5* plants, we investigated the mechanism further.

### 3.4 Grafting to determine localization (shoot or root) of *bam2* suppression of *sunn-5* phenotype

**Figure 4:**
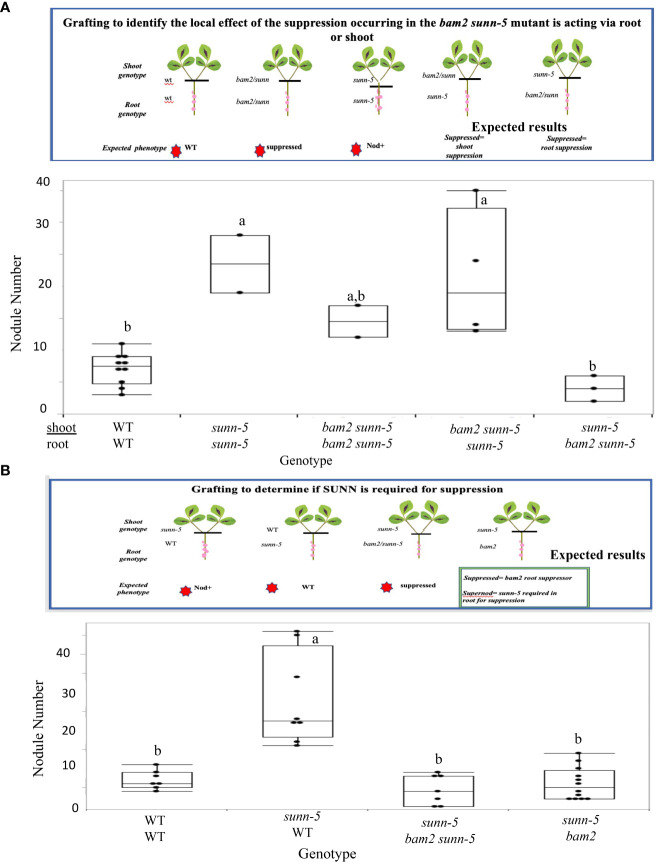
Suppression of the *sunn-5* phenotype by *bam2* is root derived and occurs regardless of *sunn-5* root genotype. **(A)** Nodule numbers formed on grafted plants to test root/shoot origin of suppression. Gene on top indicates shoot genotype; bottom indicates roots. Results are combined from two individual experiments. Box plot mean is indicated with a line (where more than three data points available), while whiskers are ± standard error, pairwise comparison Tukey–Kramer honestly significant difference (HSD). Letters indicate significance comparisons. p-value a:a,b *>* 0.01. p-value a:b *>* 0.001. **(B)** Nodule numbers formed on grafted plants to determine if SUNN is required. Experimental details are the same as in **(A)**.

The *sunn* mutation has been shown to increase nodule number when a *sunn* mutant shoot is grafted onto wild-type shoots (Penmetsa et al., 2003). The same grafting experiment was employed to determine the location of the suppression observed in the *bam2 sunn5* double mutant. Since AON is a long-distance root to shoot to root regulatory pathway, a set of grafting experiments was designed to determine the location of the effect (**Figure 4**). Comparing the nodule number of plants created with a *bam2 sunn-5* shoot grafted onto a *sunn-5* root to that of a *sunn-5* shoot on a *bam2 sunn-5* root (**Figure 4A**), suppression of the *sunn-5* hypernodulation phenotype occurred when a *sunn-5* shoot genotype was grafted onto the *bam2 sunn-5* root genotype, indicating that the suppression is root derived (**Figure 4A**). Since the root is a double mutant, two possibilities arise—either *sunn-5* is required for the suppression, or *bam2* alone in the roots suppresses the nodule number phenotype of a *sunn-5* shoot. To resolve this, a second grafting experiment was designed (**Figure 4B**) in which the *sunn-5* shoot genotype was grafted onto a *bam2 sunn-5* root genotype and compared to a *sunn-5* shoot genotype grafted onto a *bam2* root genotype. Suppression of the *sunn-5* hypernodulation phenotype occurred in both graft combinations, suggesting that *bam2* can suppress nodule numbers from the roots, with or without *sunn-5* being present (**Figure 4B**).

### 3.5 Overexpression of *BAM2* increases nodulation in wild-type roots

**Figure 5:**
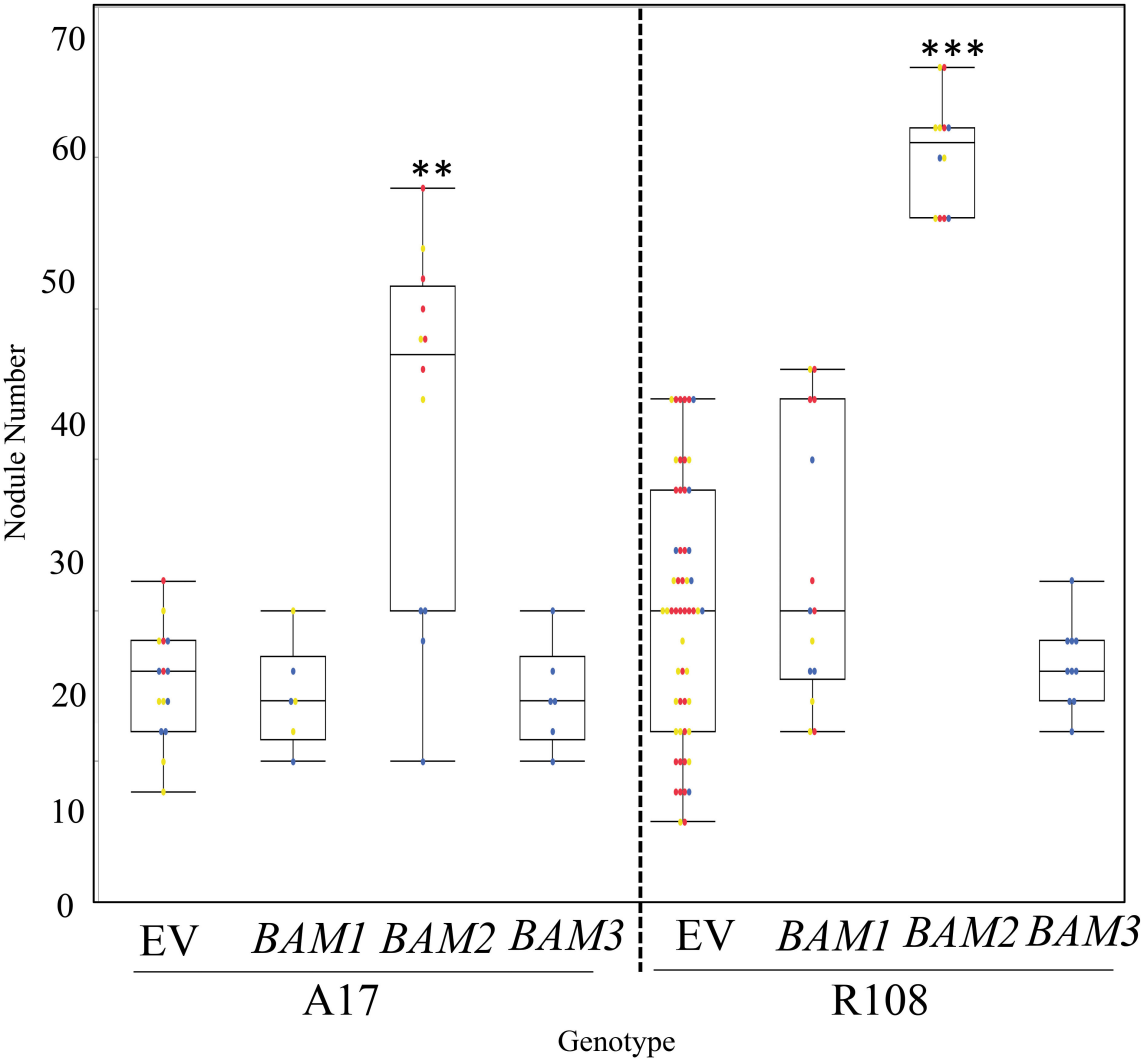
Overexpression of *MtBAM2* in wild-type A17 and R108 ecotypes increases nodule number. Nodule number in plants overexpressing *BAM1*, *BAM2*, or *BAM3* under the 35S promoter (see Materials and Methods) at 14 dpi. A17 ecotype plants are inoculated with *Sinorhizobium medicae* ABS7, and R108 ecotype plants with *Sinorhizobium meliloti* RM41. EV = empty vector control. Dots represent individual plants. Each dot color is associated with independent biological replicates: rep1 = red, rep2 = yellow, and rep3 = blue. Box plot mean is indicated with a line, while whiskers are ± standard error, pairwise comparison Tukey–Kramer honestly significant difference (HSD), p-value *>* 0.01. (**). p-value *>* 0.001. (***) denotes significance compared to EV control. Pictures of representative plants are in **Supplementary Figure 4**.

In addition to phenotyping *bam* mutants, we observed nodule numbers in transgenic hairy roots overexpressing individual *MtBAM* genes driven by the CMV 35S promoter compared to control plants carrying empty vector constructs (**Figure 5**). Overexpression of *BAM1* and *BAM3* did not alter nodule numbers in wild-type plants of either ecotype, but overexpression of *BAM2* in wild-type transgenic hairy roots caused a significant increase in nodule number in both A17 (the ecotype used for genome sequencing) and R108 (an ecotype used for tissue culture-based transformation and the background of the *BAM* mutant lines). This confirms the specific involvement of *BAM2* in nodule number and that the effect is not ecotype specific.

### 3.6 The effect of overexpression of *BAM2* in a *sunn* mutant background is allele specific

**Figure 6:**
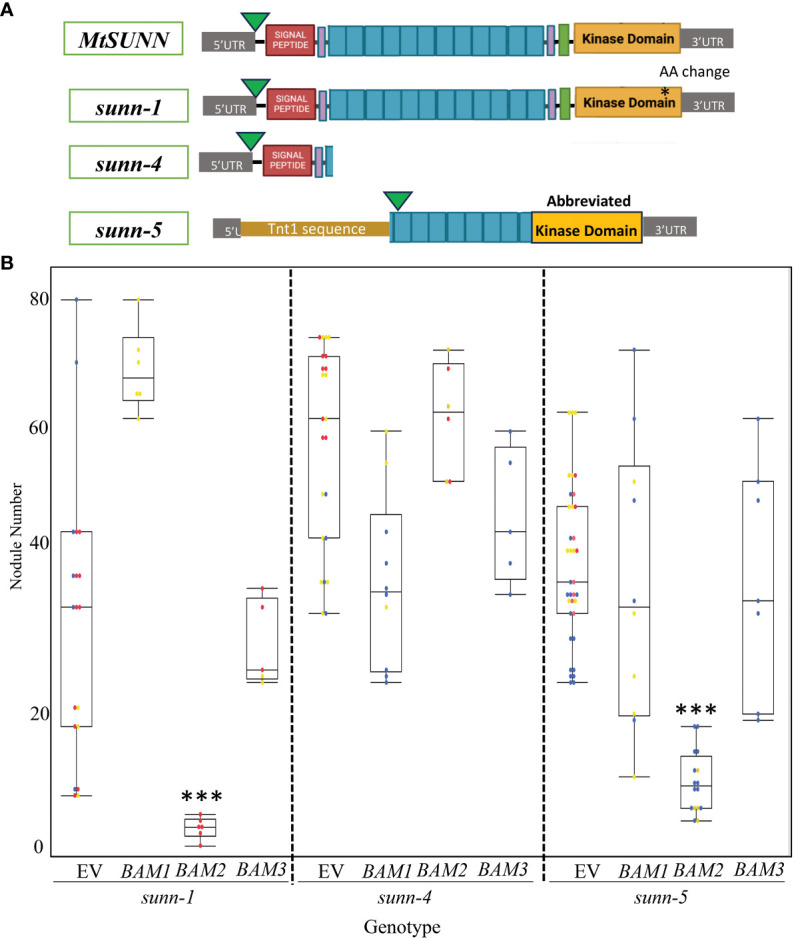
Results of overexpression of *MtBAM1*, *MtBAM2*, and *MtBAM3* in *sunn* mutants are allele specific. **(A)** Diagram of SUNN protein and effects of *sunn* alleles. Green arrow indicates Met used for start, purple boxes are paired Cys residues, turquoise boxes represent leucine-rich repeats (not to scale; there are 22), and green box is transmembrane domain. **(B)** Nodulation on plants overexpressing indicated *BAM* genes. EV = empty vector control. Dots represent individual plants. Each dot color is associated with independent biological replicates: rep1 = red, rep2 = yellow, and rep3 = blue. Box plot mean is indicated with a line, while whiskers are ± standard error, pairwise comparison Tukey–Kramer honestly significant difference (HSD). (***) denotes significance compared to EV, p-value *>* 0.001. Pictures of representative plants are in **Supplementary Figure 4**.

Suppression of nodule number in *sunn-5* could arise from disruption of signaling downstream of SUNN, or it could arise from disruption of a signaling step at which BAM and SUNN interact (Meneely, 2020). We tested an allelic series of *sunn* mutants for indications of interaction between the proteins. The *sunn-5* mutant in the R108 background has been used in previous work (Crook et al., 2016; Nowak et al., 2019) and contains a *Tnt1* insertion (~2 kb in size) located 406 bp upstream of the ATG start codon in *SUNN* gene (El-Heba et al., 2015). Using PCR on cDNA (**Supplementary Figure 1**), we determined that this promoter insertion also removed 584 bases past the start site, resulting in a potential new start in the leucine-rich repeats, as well as a deletion of the end of the repeats, the transmembrane domain and into the kinase domain (**Figure 6A**); the ability to isolate RNA indicates that a truncated *SUNN* message is made. Additional *sunn* mutants exist in the A17 ecotype; the *sunn-1* allele in A17 produces a full transcript with only a change in an amino acid in the kinase domain, while the *sunn-4* allele contains a premature stop codon immediately after the signal sequence and is considered a null allele (Schnabel et al., 2005). The two alleles vary in nodule number; *sunn-1* plants have three to five times the nodules of wild-type plants, while *sunn-4* plants have a 10-fold increase in nodule number (Schnabel et al., 2010). A17 is difficult to cross with R108 due to multiple rounds of R108 selection for regeneration and differences in the genomes, which make genetic crosses problematic. Adding to the difficulties, the R108 ecotype nodulates best with a different strain of rhizobia (Hoffmann et al., 1997). Therefore, rather than making the mutants to test suppression, we chose to overexpress *BAM2* in the different alleles of *sunn* in their respective ecotypes, inoculated with the rhizobia best for each ecotype. In contrast to wild-type plants in which overexpression of *BAM2* increased nodule number, overexpression of *BAM2* in a *sunn-5* background suppressed nodule numbers to a wild-type level (**Figure 6B**). Overexpression of *BAM2* also suppresses the hypernodulation phenotype of a *sunn-1* mutant, which produces a full-length *sunn* message with a single amino acid change (Schnabel et al., 2005) but did not suppress hypernodulation in *sunn-4* null allele (**Figure 6B**). When *BAM1* and *BAM3* were overexpressed in the *sunn-4* and *sunn-5* mutants, no alteration to the hypernodulating *sunn* phenotype was observed for either mutant (**Figure 6B**), again confirming the effect is also specific to the *BAM2* gene. The s*unn-1* mutant appeared to increase nodule number in response to *BAM1* overexpression, but this was not statistically significant because of the distribution spread of nodule number in the empty vector control.

### 3.7 Overexpression of *BAM*s in other AON mutants

**Figure 7:**
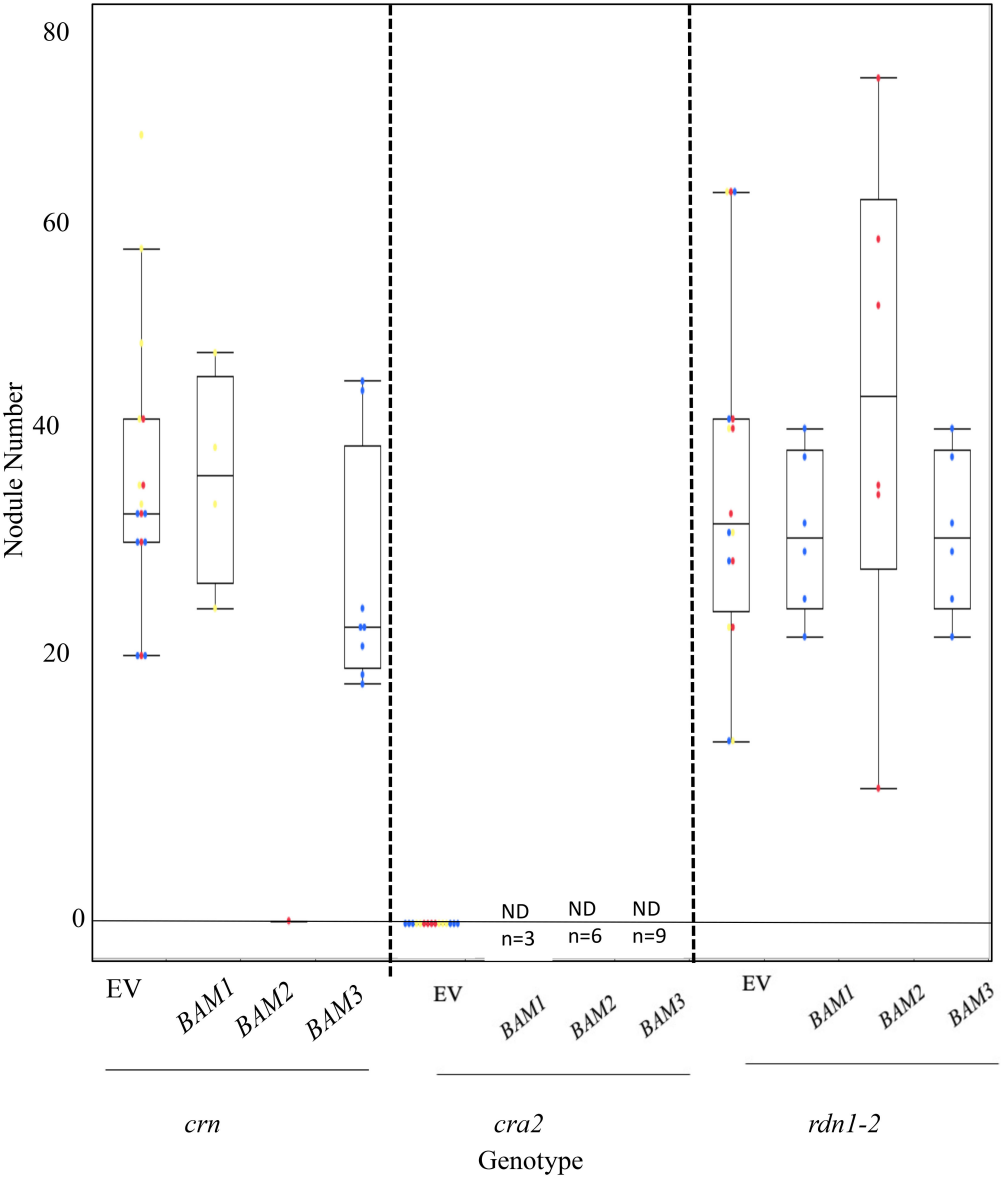
Results of overexpression of *MtBAM1*, *MtBAM2*, and *MtBAM3* in autoregulation of nodulation (AON) mutants *crn* and *rdn1-2* and *cra2* mutants. No significant nodule number phenotype was observed from the overexpression of *MtBAM1*, *MtBAM2*, or *MtBAM3* in any of the AON mutants. However, *MtBAM2* overexpression in *crn* resulted in almost all plants dying, with only one plant surviving the screening stage. No nodules formed on the sole surviving *crn*:p*MtBAM2* plant. All *cra2* plants overexpressing a *BAM* gene died before screening. EV = empty vector control. Dots represent individual plants. Each dot color is associated with independent biological replicates: rep1 = red, rep2 = yellow, and rep3 = blue. Box plot mean is indicated with a line, while whiskers are ± standard error.

To determine if *BAM2* overexpression can suppress nodulation in other hypernodulators, we repeated the previous experiment with three mutants in different portions of the AON pathway. *RDN1* functions in the root early in the AON pathway upstream of *SUNN*, modifying the ligand for the SUNN receptor (Kassaw et al., 2017). The pseudokinase CRN displays an increase in nodule number when mutated and acts at the same point as SUNN in the AON pathway, forming heteromultimers with SUNN (Crook et al., 2016). In contrast, the CRA2 receptor kinase mutants signal in a different part of nodule number regulation, separate from SUNN, conveying information on nitrogen status to the AON pathway through signaling with CEP1 peptide (Laffont et al., 2019). The *cra2* mutant does not make nodules because the signal for nitrogen needs is not sent to the roots. None of the *BAM*s affected the hypernodulation phenotype of *rdn1-2* mutants when overexpressed; however, BAM2 overexpression suppressed the hypernodulation phenotype of *crn* mutants (**Figure 7**). Interestingly, overexpression of all *BAM*s tested resulted in the death of all *cra2* plants before nodulation could occur, while their empty vector controls remained alive (**Figure 7**), suggesting that there may be some effect unrelated to nodulation of excess *BAM2* in these plants.

### 3.8 Expression of genes downstream of *BAM/SUNN*

**Figure 8:**
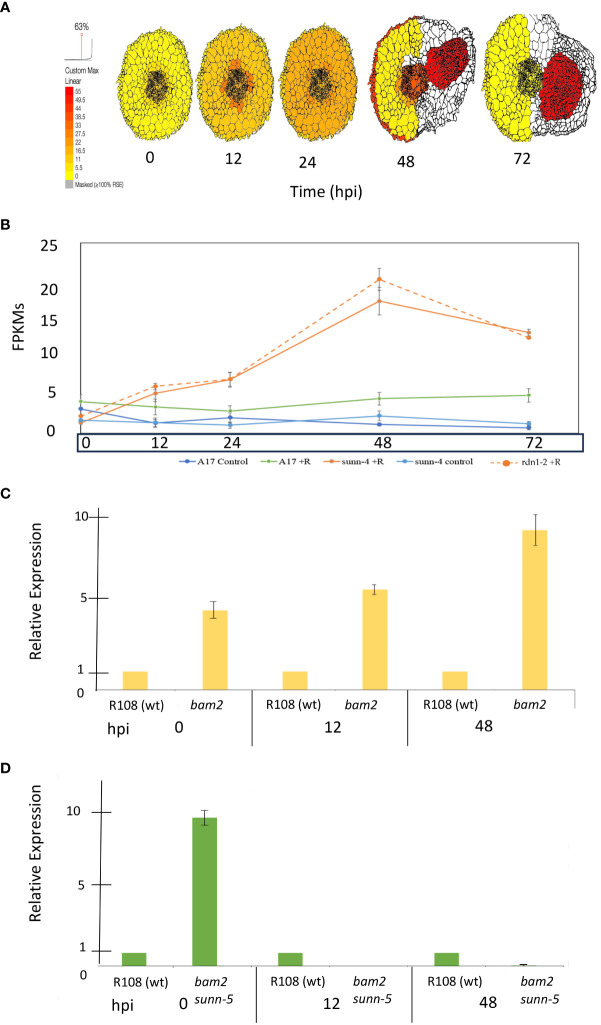
Expression of *MtWOX5* during nodule formation. **(A)** *MtWOX5* tissue-specific expression created using ePlant and the data in Schnabel et al. (2023b). **(B)** Whole root expression traces for *WOX5* from the same time course and conditions were taken from the data in Schnabel et al. (2023a). Expression (FPKMs) of *WOX5* from 0 to 72 hpi is displayed as blue = A17 control, green = A17 + rhizobia, light blue = *sunn-4* control, orange = *sunn-4* + rhizobia, and orange dotted = *rdn1-2* + rhizobia. **(C)** Expression of *MtWOX5* in *bam2* mutants and **(D)** *bam2 sunn-5* mutants. For each time point, relative expression was calculated by fold change compared to the wild-type control at that time point (see Materials and Methods). Data represent three biological and three technical replicates. Error bars represent ± standard error. Note that *WOX5* expression was undetectable in the *bam2 sunn-5* mutant at 12 hpi and barely detectable at 48 hpi.

The *MtWOX5* transcription factor is downstream of *SUNN* signaling. Since *MtWOX5* has been implicated in nodule development in *M. truncatula* (Osipova et al., 2012), in this work, we examined expression levels in several mutant combinations. We examined the relative expression of *MtWOX5* during nodulation in both *bam2* and *bam2 sunn-5* plants by utilizing a time-course experiment using root segments to assess the relative expression of each marker gene at 0, 12, and 48 hpi. Previous work in our lab measured absolute expression for each gene in wild-type and *sunn-4* root segments, and we also used the ePlant resource to confirm the tissue-level expression of these genes. We examined the expression of *MtWOX5* at each time point in wild-type, *bam2*, and *bam2 sunn-5* plants using quantitative real-time PCR (**Figure 8**).

*MtWOX5* expression is the strongest in developing nodules (**Figure 8A**) and may reflect the expression of the nodule meristem since the LCM resource colors the entire tissue. *MtWOX5* maintains a steady low-level expression throughout nodule development but is slightly higher in the inoculated roots compared to the non-inoculated controls (**Figure 8B**). In *sunn-4* roots, *MtWOX5* expression increases at 12 hpi and continues to increase to peak expression in this time course at 48 hpi (**Figure 8B**). In contrast, in a *bam2* background, *WOX5* expression as measured using real-time quantitative RT-PCR is greater at 0, 12, and 48 hpi compared to the wild-type control (**Figure 8C**). However, in the double-mutant *bam2 sunn-5* root segments, the highest level of relative expression of *MtWOX5* was observed at 0 hpi, with expression less than that of the wild-type control at 12 and 48 hpi in the double mutant (**Figure 8D**). Expression of the master regulator *MtWOX5* decreases after inoculation in the *bam2 sunn-5* mutant versus in the single *bam2* background in which *MtWOX5* increases and stays up after inoculation.

## 4 Discussion and conclusions

Phylogenetic analysis (**Figure 1**) suggests the expansion in the *BAM* gene family between *Arabidopsis* with three *BAM* genes, and *M. truncatula* with five suggests that if the expansion of the gene family resulted in new genes used for nodule meristems, *MtBAM4* and *MtBAM5* would be the best candidates based on their position with additional legume *BAM*s in a clade away from most of the rest of the *BAM*s. However, neither *BAM4* nor *BAM5* was expressed in nodules (**Figure 2**), while *MtBAM1–3* were expressed in nodules.

Since we did not identify nodule number or root length phenotype in any plants carrying a mutation in a single *bam* (**Supplementary Figure 2**) or any of the plants carrying two *bam* mutations (**Supplementary Figure 3**), mutation analysis of *BAM* genes alone did not identify a *BAM* specific to the regulation of nodule number. The lack of an observable effect from single *bam* mutants was not unexpected, as single *Atbams* showed no phenotype (DeYoung et al., 2006). While we did not investigate further, *MtBAM1* could be important to multiple developmental systems, as we were unable to generate any double mutants with this line. The discovery that adding a *bam2* mutation to a *sunn* mutation suppressed nodule number to wild-type levels (**Figure 3**) indicates *BAM2* is involved in nodule development, but visualization of this role is only possible when *SUNN* is disrupted. This effect is specific to *bam2* and not observed when *sunn-5* is combined with *bam3* or *bam5*. We were unable to test the mutations of *bam4* or *bam1*, as a double mutant with *sunn-5* could have a suppressive effect; however, *bam4* is not expressed in nodulating roots, making suppression unlikely. Since overexpression of *BAM2*, but not *BAM1* or *BAM3*, increases nodule number in wild-type plants (**Figure 5**), multiple lines of evidence support a specific role for *BAM2* in nodule number regulation.

The data support the action of BAM2 in nodulation as root specific rather than systemic. While SUNN signals from shoot to root to control nodule number (Penmetsa et al., 2003) and is central to AON (reviewed in Roy et al., 2020), *SUNN* is expressed throughout the plant in the vasculature (Schnabel et al., 2012), and the function of SUNN in the root has not yet been determined. The localization of the suppressor effect of *bam2* in the root (**Figure 4A**) is particularly interesting, as the SUNN protein is a systemic negative regulator of nodule number; *sunn-5* mutants hypernodulate because they cannot send a wild-type regulation signal to the roots to control nodule number. Nevertheless, the mutation in *bam2* in the roots of a *sunn-5* mutant allows the regulation to proceed, suggesting that the lack of *bam2* compensates for the lack of the SUNN signal from the shoots (**Figure 4B**). Further support of a local rather than systemic effect is the grafting experiment in which a *bam2* mutant in the shoot but not the root does not suppress the *sunn* hypernodulation phenotype (**Figure 4A**). All *sunn* mutants have normal nodulation if mutant roots are grafted to wild-type shoots (Penmetsa et al., 2003; Schnabel et al., 2010, **Figure 4B**), suggesting that whatever the function of SUNN in the root, a mutation of *sunn* in the root is not causal to hypernodulation. However, the addition of a *bam2* mutation in the root along with a *sunn* mutation changes the response to the shooting signal, supporting action locally in the root versus systemic action.

In *Arabidopsis*, the differentiation of stem cells in the root apical meristem and cell division in the root is controlled by CLE peptides (Willoughby and Nimchuk, 2021). Recent studies have shown that the SUNN ortholog CLV1 signals through a BAM protein complex to control CLE-mediated signaling of the root apical meristem (Wang et al., 2022) and root phloem development (Hu et al., 2022). Nodule primordia use many of the same regulatory genes, as lateral root primordia (Schiessl et al., 2019) contain a nodule meristem and develop vasculature at the time points used to measure gene expression in this work (48–72 hpi in our growth systems), making the involvement of a BAM quite likely.

In our system, both the *sunn-5* and *bam2* mutants make transcripts detectable by PCR of cDNA, but *sunn-5* is not likely to produce protein due to the lack of regulatory sequences around the possible alternate start in *sunn-5* (**Figure 6**), while the disruption of the coding sequence in all of the *bam Tnt1* insertion mutants occurs from insertion of the *Tnt-1* sequence in the middle of the LRR region, leading to a truncated protein (**Supplementary Figure 1**). In *Arabidopsis*, truncated BAM proteins lacking a kinase domain interfere with meristem homeostasis in a dominant negative manner because of the LRR repeats interacting with other proteins (Wang et al., 2018), but this is not observed in our system, suggesting that the insert in the LRR disrupts any dominant negative effect from the lack of a kinase domain.

The expression of *BAM2* in wild-type plant roots before nodulation (0 hpi in **Figure 2**, right panel) is approximately half the expression of *BAM2* in *sunn-4* mutant roots and *rdn1-2* hypernodulation mutant roots before nodulation begins. *BAM2* expression rises in roots of all inoculated plants over the 72-hpi time course, but the rise is larger in the hypernodulation mutants *sunn-4* and *rdn1-2*, and there is no reason not to expect the same pattern in *sunn-1* and *sunn-5* mutants given that the nodule number phenotypes of these mutants are similar to *rdn1-2*. If increased *BAM2* expression is observed in plant roots that hypernodulate, it is logical that overexpression of *BAM2* in wild-type roots increases nodule number. More interesting is the observation that adding more *BAM2* to *sunn* mutant roots already expressing higher levels of *BAM2* than wild-type decreases nodule number for *sunn-1* and *sunn-5* mutant roots but not *sunn-4* roots. The decrease in the *sunn-1* and *sunn-5* roots could be explained if the level of *BAM2* from the overexpression construct rises high enough to trigger RNA interference, acting like a *bam2* mutation, but the lack of suppression in *sunn-4* roots is perplexing. Because the expression of *BAM2* in these roots was not measured, it is not possible to rule out the explanation that the construct was not correctly expressed. However, the effect was seen in six plants tested in two independent experiments, giving confidence in the result. Since the transformations are performed in hairy roots in which auxin homeostasis is perturbed, it is possible that the extreme hypernodulation phenotype in the null allele is affected more by the hairy root environment than expression of *BAM* genes, given that the range of nodule numbers in **Figure 6** is below the 125 average nodules per plant in *sunn-4* mutants on an aeroponic system in all conditions including the empty vector (Schnabel et al., 2010), adding difficulties to interpretation of the *sunn-4* results.

It is important to note that overexpression of wild-type *BAM2* did not suppress (or enhance) hypernodulation in the *crn* and *rdn1-2* AON mutant roots, even though the *rdn1-2* mutant has high *BAM2* expression compared to wild type, so the effect of the mutation is not on the AON pathway itself: the suppression seems to be specific to plant roots carrying mutations in *SUNN*, with *sunn-4* as an exception. Relative expression measurements of *MtWOX5* add some insight into how the downstream nodulation signaling may be disrupted in *bam2* and *bam2 sunn-5* mutants. *MtWOX5* expression in the nodule apical meristem (NAM) (Osipova et al., 2011; Roux et al., 2014) provides molecular support for the derivation of nodule development programs from lateral root developmental programs (Hirsch and Larue, 1997; Mathesius et al., 2000; Bright et al., 2005; Desbrosses and Stougaard, 2011). *WOX5* gene is downstream from the CLE/CLV1/BAM signaling complex in *Arabidopsis* (Willoughby & Nimchuk, 2021), but other receptor kinases are involved in connecting CLE/CLV1/BAM signaling to WOX5 expression (Wang et al., 2022). The expression of *MtWOX5* is increased compared to the wild type at 0 hpi *bam2* mutants and *bam2 sunn-5* mutants but is the same as the wild type in *sunn-4* and *rdn1-2* hypernodulation mutants (**Figure 8**). In contrast to the steady higher expression observed in *sunn-4*, *rdn1-2*, and *bam2* mutants at 12 and 48 hpi, the *bam2 sunn5* mutant decreases *MtWOX5* expression below wild-type levels. Rather than eliminating nodule development, the lower expression is correlated with only a reduction of nodule number in *sunn-5* mutants. Likewise, even though *bam2* mutants have increased *MtWOX5* expression, the number of nodules is not affected, suggesting that other factors outside of *MtWOX5* are also involved in nodule number regulation.

In the event of genetic or environmental disruption, plants can initiate a genetic buffering mechanism called “active compensation” in which genes change their behavior to compensate for the disruption (Rodriguez-Leal et al., 2019). For example, in *Arabidopsis*, the weak *clv1* phenotype is genetically buffered by the paralogous BAM receptors through active compensation (Diss et al., 2014; Nimchuk et al., 2015). The loss of *CLV1* resulted in an increased expression of BAMs and a change in their expression domains, allowing for the compensation of *CLV1* loss (Nimchuk et al., 2015). In *Solanum lycopersicum*, the loss of *SlCLV3*, the tomato CLV3 ortholog, triggers an active compensation mechanism, by which the upregulation of *SlCLE9* buffers stem cell homeostasis in tomato (Rodriguez-Leal et al., 2019). Considering the related distance between *Arabidopsis* and tomato, genetic buffering of stem cells reflects a determining feature of indeterminate meristem development, including nodule meristems (Rodriguez-Leal et al., 2019). A similar effect may be happening with *BAM2* and *SUNN*.

We speculate that the loss of, or disruption to, the CLE/SUNN/BAM2 signaling in the roots alters signaling, affecting the nodule-specific transcription factor *MtWOX5*. We propose a genetic model, wherein the specific root interactions of BAM2/SUNN are critical for signaling in nodule meristem cell homeostasis in *M. truncatula*. Except for the perplexing *sunn-4* overexpression results, an anomaly to pursue in future studies, our data support the involvement of CLE/CLV1/BAM signaling influencing nodule number in *M. truncatula*.

## Data availability statement

The original contributions presented in the study are included in the article/**Supplementary Material**. Further inquiries can be directed to the corresponding author.

## Author contributions

JT: Data curation, Formal analysis, Investigation, Methodology, Writing – original draft, Writing – review & editing. JF: Conceptualization, Formal analysis, Funding acquisition, Methodology, Resources, Supervision, Visualization, Writing – review & editing.
