# Supplementary figures and images for: Mutation of BAM2 rescues the sunn hypernodulation phenotype in Medicago truncatula, suggesting that a signaling pathway like CLV1/BAM in Arabidopsis affects nodule number
Source: Front Plant Sci. 2024 Jan 11;14:1334190. doi: 10.3389/fpls.2023.1334190 (PMC10808729; doi:10.3389/fpls.2023.1334190)

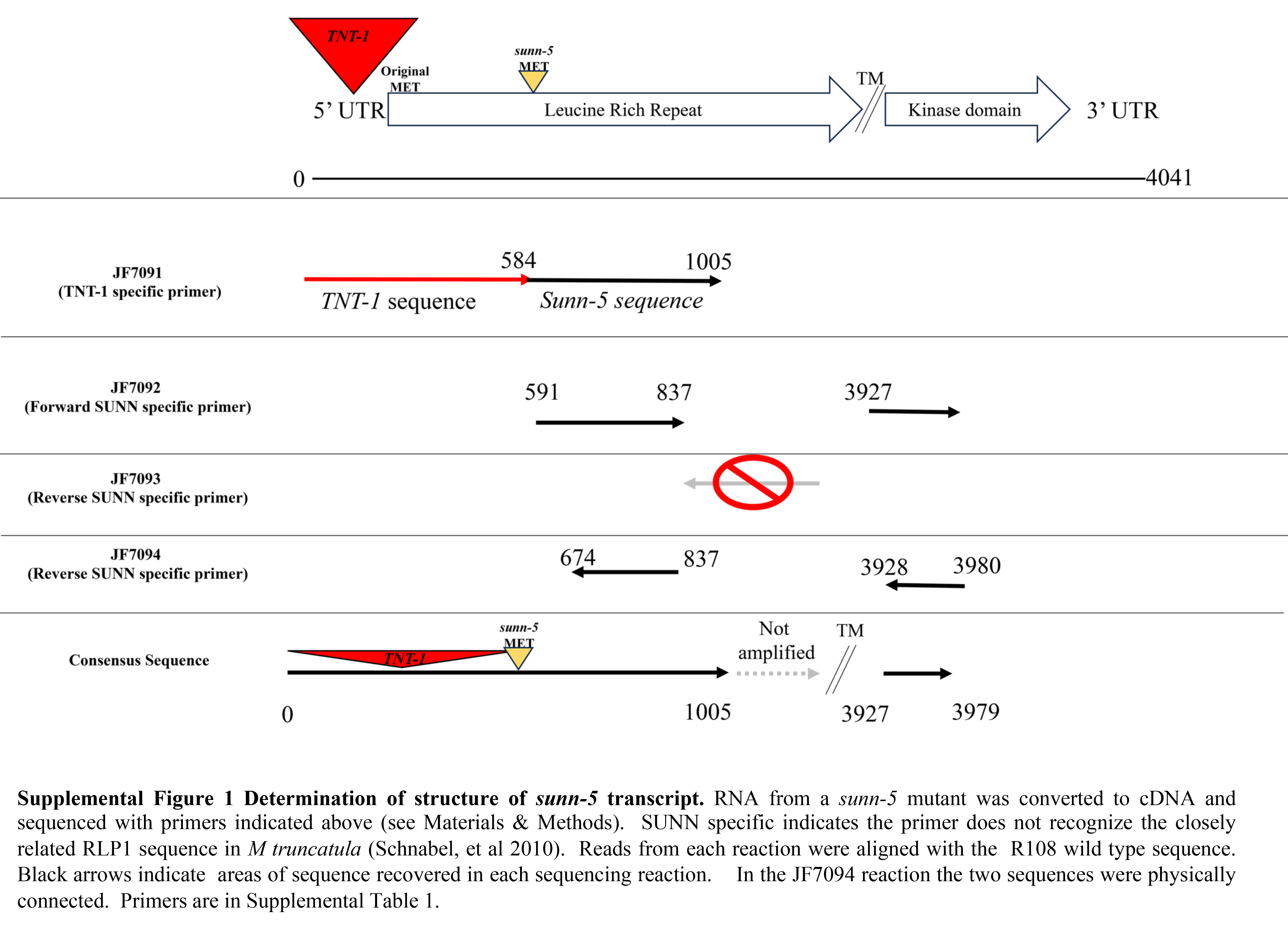

Supplement: Supplementary file 1 [file Image_1.tif]

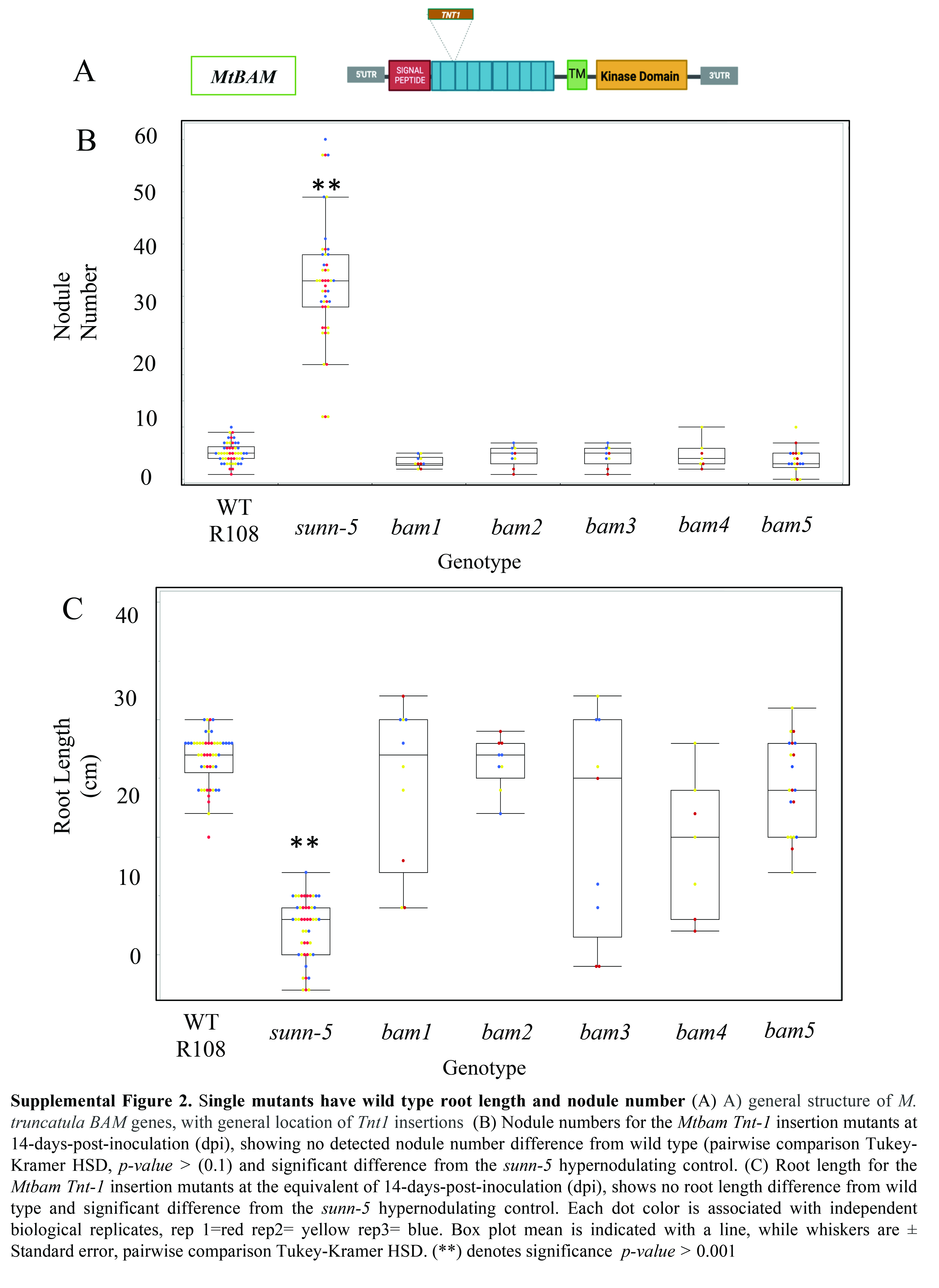

Supplement: Supplementary file 2 [file Image_2.tif]

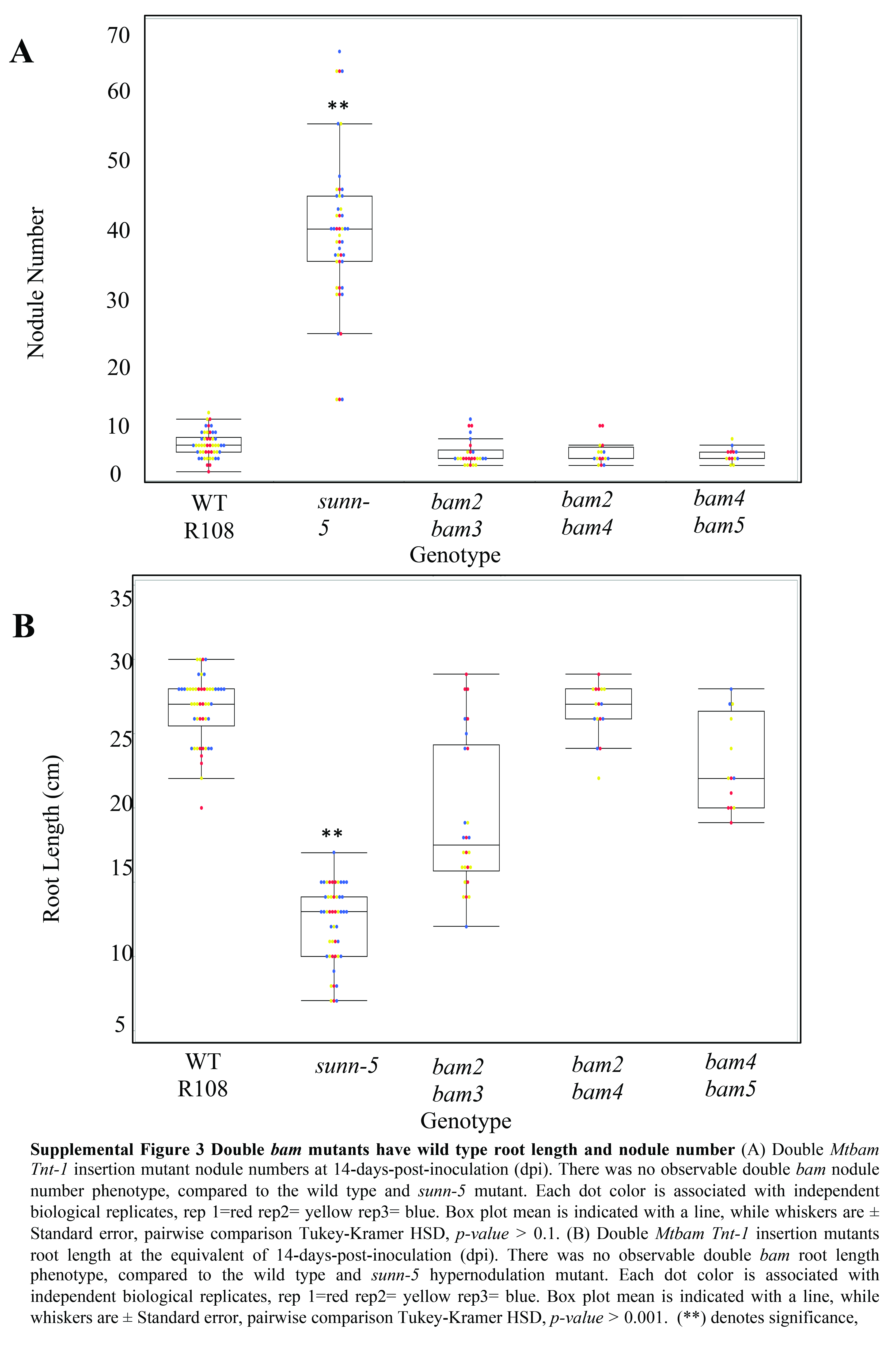

Supplement: Supplementary file 3 [file Image_3.tif]

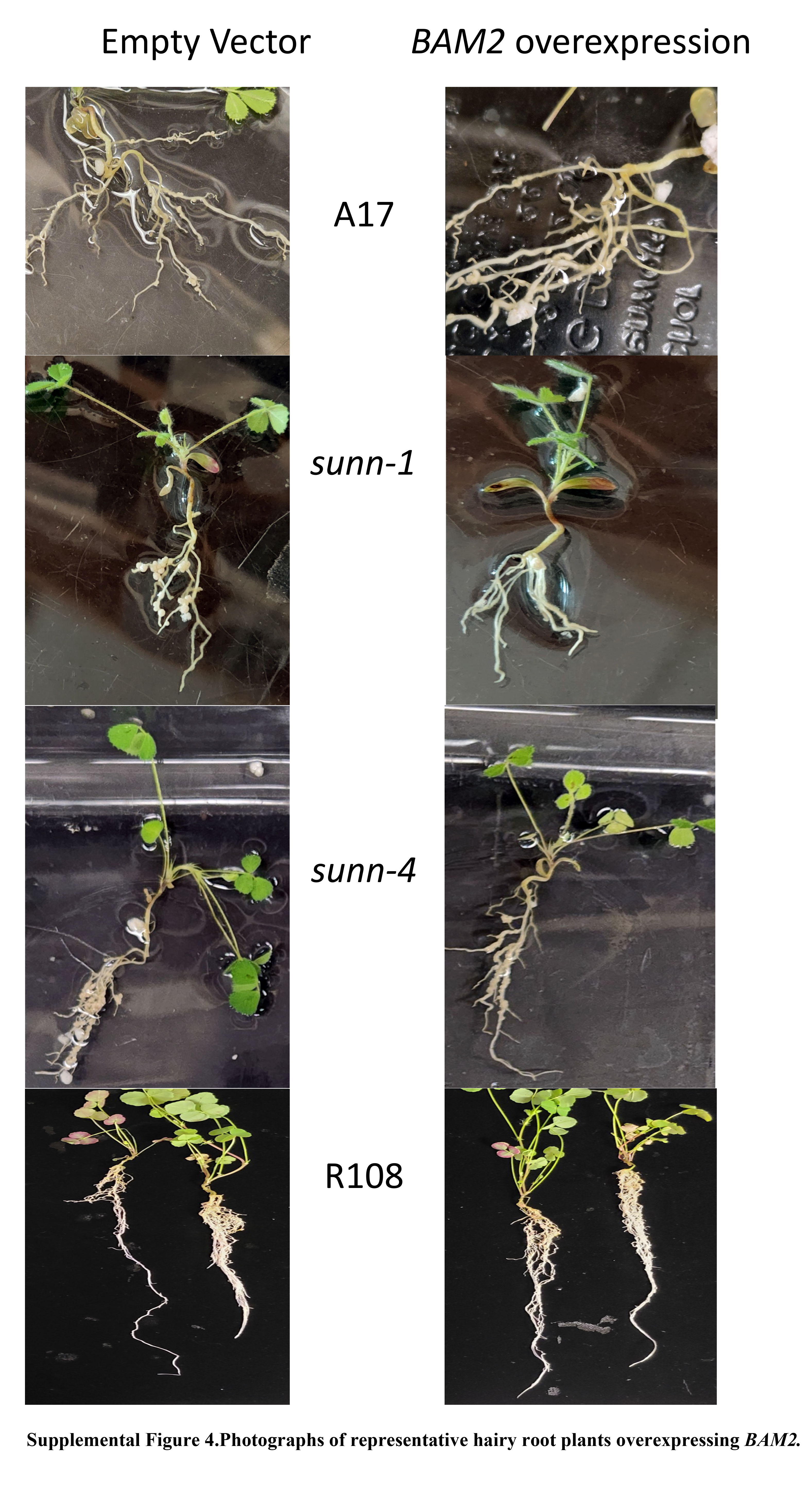

Supplement: Supplementary file 4 [file Image_4.tif]
