# Supplementary material for: Mutation of BAM2 rescues the sunn hypernodulation phenotype in Medicago truncatula, suggesting that a signaling pathway like CLV1/BAM in Arabidopsis affects nodule number
Source: Front Plant Sci. 2024 Jan 11;14:1334190. doi: 10.3389/fpls.2023.1334190 (PMC10808729; doi:10.3389/fpls.2023.1334190)
Supplement: Supplementary file 5 [file Table_1.docx]

| Supplemental Table 1 | Genes used in phylogenetic analysis tree |
| --- | --- |
|  |  |
| Name of gene in table | Accession number of sequence |
| MtSUNN | Medtr4g070970 |
| MtBAM3 | Medtr5g090100 |
| MtBAM2 | Medtr5g014700 |
| MtBAM1 | Medtr4g097880 |
| MtBAM4 | Medtr3g449390 |
| MtBAM5 | Medtr2g005810 |
| BAM1 | At5g65700 |
| BAM2 | At4g20270 |
| BAM3 | At3g49670 |
| CLV1 | AT1G75820 |
| PvNARK | AAN74865.1 |
| PvBAM3 | Phvul006G029000 |
| PvBAM1/2B | Phvul003G231400 |
| PvBAM1/2A | Phvul003G231350 |
| PvBAM2 | Phvul002G007600 |
| LjHAR1 | Lj3g3v3375780 |
| LjSer-Thr-LRR6 | Lj6g3v2275150 |
| LjSer-Thr-LRR1 | Lj6g3v0937000 |
| LjSer-Thr-LRR3 | Lj3g3v1933090 |
| LjSer-Thr-LRR4 | Lj2g3v3058590 |
| LjSer-Thr-LRR2 | Lj2g3v1984380 |
| PsSYM29 | AJ495759 |
| PsBAM1/2B | XP050906926.1 |
| PsBAM1/2A | XP050871590.1 |
